# The Role of Novel Biomarkers of Cardiovascular Disease in Chronic Kidney Disease: Focus on Adiponectin and Leptin

**DOI:** 10.2174/157340308786349516

**Published:** 2008-11

**Authors:** Omar M Kaisar, David W Johnson, Judith B Prins, Nicole Isbel

**Affiliations:** 1 Department of Nephrology, University of Queensland, Princess Alexandra Hospital, Brisbane, Queensland, Australia; 2 Diamantina Institute for Cancer, Immunology and Metabolic Medicine University of Queensland, Princess Alexandra Hospital, Brisbane, Queensland, Australia

**Keywords:** Biomarker, cardiovascular disease, adiponectin, leptin, kidney disease.

## Abstract

Cardiovascular disease (CVD) remains a major cause of premature death in patients with chronic kidney disease (CKD), including renal transplant recipients. Both interplay of traditional cardiovascular and renal specific risk factors have been shown to be associated with an increased risk of cardiovascular death in patients with CKD. Recently, there has been great interest in the role of novel biomarkers, in particular adiponectin and leptin, and its association with CVD in the CKD population. Adiponectin is a multifunctional adipocyte-derived protein with anti-inflammatory, antiatherogenic and insulin sensitizing activity. Recent observational studies have shown adiponectin to be a novel risk marker of CVD in patients with stages 1 to 5 CKD. Leptin is an adipocyte-derived hormone that promotes weight loss by decreasing food intake. Similarly, there are observational studies to support an association between leptin and CVD, including patients with CKD. In the CKD population, leptin may be associated with uremic cachexia and subsequent increased mortality. This review aims to summarize the pathophysiological and potential clinical roles of these cardiovascular biomarkers in patients with CKD.

## INTRODUCTION

Cardiovascular disease (CVD) remains the major cause of premature death in patients with chronic kidney disease (CKD). CKD increases the risk of cardiac death 10-20 fold compared to the non-CKD population despite stratification for age, sex, race and diabetes [1, 2]. The two predominant clinical presentations of CVD in CKD are left ventricular hypertrophy (LVH) and coronary artery disease (CAD) [3]. Furthermore, the risk of cardiovascular disease and cardiac death rises significantly with glomerular filtration rate (GFR) under 60mls/min and is incremental with further declines in kidney function [4, 5]. In fact, the majority of patients with CKD die from CVD prior to reaching end stage kidney disease (ESKD) [6]. Both an interplay of traditional cardiovascular and renal specific risk factors (anemia, oxidative stress, inflammation, hyperhomocysteinemia) have been shown to be associated with an increased risk of cardiovascular death in patients with CKD. Despite, the negative impact of CVD in patients with CKD, various randomized controlled studies have either excluded patients with kidney disease or have failed to show any benefit in the treatment of either traditional or renal specific risk factors on cardiovascular outcome [7-9]. In addition, recent epidemiological studies have shown obesity and the metabolic syndrome to be independent predictors for CKD and progression to ESKD [10, 11]. As such, there has been a growing interest in the role of novel biomarkers and its association with CVD in patients with CKD, in particular the adipokines adiponectin and leptin. This review aims to summarize the pathophysiological and potential clinical role of these adipokines in patients with CKD. 

## ADIPONECTIN

In recent years, there has been a greater appreciation of adipose tissue as an active metabolic organ beyond a storage depot for triglycerides, including the secretion of various adipokines, such as adiponectin and leptin. Human adiponectin is a multifunctional adipocyte-derived protein with anti-inflammatory, anti-atherogenic and insulin sensitizing activity [12]. It is the most abundant adipocyte-derived protein, with a collagenous N-terminal and a globular carboxyl terminal with sequence homology to the C1q family of complement proteins and collagen X and VIII. It circulates in low, medium and high molecular weight forms with clinical data indicating that the high molecular weight form most strongly correlates with improved insulin sensitivity and glucose tolerance as well as having protective anti-atherosclerotic effects [13, 14]. Adiponectin levels have been shown to be lower in males, obesity, insulin resistance (including metabolic syndrome), type 2 diabetes mellitus, coronary artery disease and essential hypertension [15-18]. In contrast, CKD is associated with hyperadiponectinemia including nephrotic syndrome [19]. This is more pronounced with more severe CKD, such that end-stage kidney disease patients have serum adiponectin levels up to three times higher than those in the normal population [20]. The rise in adiponectin with declining glomerular filtration rate (GFR) has been postulated to be related to impaired renal elimination or metabolism, though the exact role of the kidney in the biodegradation and excretion of adiponectin remains unclear. 

### The Role of Adiponectin as a Cardiovascular Risk Factor in Chronic Kidney Disease

Hypoadiponectinemia predicts the development of CVD in the non-CKD population. For example, longitudinal follow-up of male patients with type II diabetes showed that those with the highest quintile of adiponectin compared to the lowest quintile had a lower risk of developing CAD and myocardial infarction, independent of other variables [21]. Recent observational studies have shown adiponectin to be a novel risk marker of CVD in patients with stage 1 to 5 CKD. These prospective studies have revealed that hypoadiponectinemia predicts the development of new cardiovascular events in patients with CKD, including the hemodialysis population. The inverse relationship between adiponectin, GFR and CVD in patients with CKD suggests a protective role for adiponectin against the development of cardiovascular events in this patient population. This association has been highlighted in several recent studies. Zoccali *et al.* showed that in their cohort of 227 hemodialysis patients who were followed for a mean of 31±13 months that each 1 µg/mL increase in adiponectin concentration was associated with a 3% risk reduction in new cardiovascular events [22]. The relative risk of adverse cardiovascular events was 1.56 times (95% confidence interval, 1.12 to 1.99 times) higher among patients in the first adiponectin tertile, compared with those in the third tertile. In this study, higher adiponectin levels predicted lower cardiovascular events in the hemodialysis patients independent of other variables. Analogous results have been observed in a study by Becker *et al.*, from the Mild to Moderate Kidney study database in non-diabetic subjects with primarily stage 3 to 4 CKD [measured GFR between 38 and 96mls/min] [23]. At a median follow-up of 54 months, patients who developed new cardiovascular events had significantly lower adiponectin levels (odds ratio, 0.72; 95% CI 0.58 to 0.89; P*=*0.002). Further corroborating evidence for this link has been provided in a recently published study by Iwashima *et al.*, who followed 150 patients with CKD prospectively for a mean period of 32 months [24]. Adiponectin levels were significantly higher in patients with more severe kidney impairment as assessed by CKD stage. After dividing the patients into a high and low group based upon gender-specific median values of adiponectin, the low adiponectin group had a significantly shorter event-free survival compared to the higher group (<4.39 µg/ml in men, <6.84 µg/ml in women, P<0.03). Using multivariate Cox proportional hazards model analysis, each 1 µg/ml increase in adiponectin was associated with a 14% decrease in the risk of CVD (adjusted hazard ratio 0.86, 95% CI 0.75 to 0.96, P= 0.004). Furthermore, adiponectin may be predictive of future ischemic cardiac events, as patients with previous IHD (n = 65), had a significantly shorter IHD-free survival in the lower adiponectin group (<4.45 µg/ml in men, <4.49 µg/ml in women, P<0.05). In addition, the percentage of the high-molecular-weight form of adiponectin in patients with severe CKD and IHD during follow-up was significantly smaller than that in those without IHD (P<0.02). 

In contrast to the above findings, Menon *et al.*, in their analysis of 820 patients with a GFR range of between 13 and 55 mls/min from the MDRD (Modified Renal Diet) database revealed a direct correlation between adiponectin and the relative risk of cardiovascular mortality [25]. In multivariable adjusted Cox models, a 1 µg/ml increase in adiponectin was associated with a 3% (hazard ratio 1.03; 95% CI 1.01 to 1.05; P=0.02) elevated risk for all-cause and 6% (hazard ratio 1.06; 95% CI 1.03 to 1.09; P=0.001) higher risk for cardiovascular mortality. In view of the strong association between CKD and CVD, the direct correlation between adiponectin and CVD in the MDRD study may be due to a residual confounding effect of reduced renal function or processes accompanying CKD, but unadjusted for reduced GFR. For example, the mean GFR and adiponectin in the MDRD study was 32 mls/min and 6 µmol/L respectively, in comparison to 63 mls/min and 12 µmol/L correspondingly in the study by Becker *et al.*, highlighting the strong association between adiponectin and GFR [20]. Furthermore, in a recent study involving hemodialysis patients who underwent coronary artery stenting for ischemic heart disease, only adiponectin in multiple logistic regression analysis was shown to be associated with increased in-stent restenosis with an odds ratio for restenosis of 0.651 (P=0.001, 95% confidence interval: 0.506–0.839) with each 1 μg/ml increase in plasma adiponectin concentration [26]. In this study, lower adiponectin levels predicted increased in-stent restenosis.

### Mechanism of Adiponectin Cardiovascular Protection

While the above observational studies have linked adiponectin to improved cardiovascular outcomes in patients with CKD, it remains unclear as to how adiponectin confers vascular protective benefits. However, there is now evolving evidence of its mechanistic actions that may account for its cardioprotective benefits.

Adiponectin is an abundant protein hormone, comprising nearly 0.01% of all plasma proteins [27]. Exclusively secreted by adipocytes, it possesses anti-inflammatory, anti-atherogenic and beneficial metabolic actions. Adiponectin improves insulin sensitivity by inhibiting hepatic gluconeogenesis and increased glucose uptake *via* translocation of the insulin responsive glucose transporter (GLUT4) to the cell surface in skeletal muscle cells [28]. Hypoadiponectinemia is also associated with dyslipidemia, in particular elevated triacylglycerol rich lipoproteins, a common feature of obesity related dyslipidemia. In humans including healthy adults and subjects with type 2 diabetes, adiponectin has been shown to be positively correlated to high density lipoproteins and inversely related to triglycerides (TG), very low density lipoproteins and low density lipoproteins [29-31]. Adiponectin may have a direct effect on improving dyslipidemia, particularly in obesity. In animals, administration of adiponectin to lipodystrophic and obese mice has been shown to reduce TG and free fatty acid levels [32].

Furthermore, adiponectin is inversely related to various inflammatory markers such as tumour necrosis factor-α (TNF-α), C-reactive protein (CRP) and interleukin-6 (IL-6) in normal subjects and patients with type 2 diabetes and CVD [33-35]. In addition, adiponectin has also been shown to exhibit anti-inflammatory activity in atherosclerotic experimental models including reduction in TNF-α production [36, 37]. Inflammatory markers are typically elevated in CKD and are predictive of cardiovascular events in this population. It is unclear if higher adiponectin levels in kidney failure represents a compensatory process to the inflammatory milieu in the CKD, as other unknown variables, particularly associated with uremia may confound this association. While direct anti-inflammatory actions may benefit the vasculature in CVD, there is evolving evidence for direct anti-atherogenic actions of adiponectin. The hallmark of atherosclerosis is proliferation and migration of vascular smooth muscle cells (VSMC). In experimental models, adiponectin ameliorates VSMC proliferation by inhibition of mitogen-activated protein kinase and other growth factors capable of stimulating smooth muscle synthesis including platelet derived growth factor and fibroblast growth factor [38]. Adiponectin has also been shown to increase production of nitric oxide and tissue inhibitor of metalloproteinase-1, resulting in vasodilatation and decrease in plaque rupture, respectively [38, 39].

While most studies to date point to a consistent link between lower adiponectin levels and CVD in patients with CKD, there are no interventional studies to demonstrate any benefit in increasing adiponectin levels on cardiovascular outcome in this group. Several studies in non-CKD patients have demonstrated increased adiponectin levels with weight loss, physical exercise and the use of pharmacological agents, including angiotensin converting enzyme inhibitors, angiotensin 2 receptor blockers, statins and thiazolidinediones [40-42]. Similarly, there have been two studies demonstrating increased adiponectin levels with the use of ramipril and candersartan in type 2 diabetics with proteinuria and normal kidney function and patients on peritoneal dialysis, respectively [43, 44]. However, to date, there have been no studies that have investigated the prognostic impact of increasing adiponectin levels on cardiovascular morbidity and mortality, regardless of the absence or presence of kidney disease.

## LEPTIN

In contrast to studies which have consistently found a strong inverse association between adiponectin and CVD, an association between leptin and CVD has been less clearly demonstrated. Leptin, a 16kDA protein is a product of the obesity (*ob*) gene, which is secreted by white adipose tissue and primarily bound to protein in lean adults [45]. Leptin forms part of the IL-6 family of pro-inflammatory cytokines and is principally cleared from the circulation by the kidney *via* a combination of glomerular filtration and subsequent tubular degradation [46]. Leptin levels are elevated in proportion to insulin levels, glucocorticoids, cytokines and particularly obesity [47]. Leptin is anorectic and in normal humans functions to decrease appetite and in mice also increases energy expenditure. Similar to adiponectin, there has been a great interest in the role of leptin in CVD. In contrast to adiponectin, leptin levels are inversely related to body fat mass [48]. The inability of leptin to induce weight loss in this instance is presumed to be related to leptin resistance. Leptin exerts its anorectic effects *via* the hypothalamus. Leptin binds to the b isoform of the obesity receptor (Ob-Rb) and increases the synthesis of proopiomelanocortin *via* the Janus-activated kinase (JAK) and signal transducers and activators of transcriptions (STAT) pathway. Proopiomelanocortin is subsequently converted to α-melanocyte-stimulating hormone, which in turn activates melanocortin-3 and -4 receptors to decrease appetite [49]. Furthermore, leptin antagonizes the actions of the appetite stimulating hormone, neuropeptide Y, and the inhibitory effects of Agouti-related protein on melanocortin-3 and -4 receptors signaling pathways [50]. The exact role of leptin resistance is ill-defined but postulated mechanisms include elevated suppressors of the cytokine signaling family (SOCS3) levels (which results in negative feedback inhibition of the JAK-STAT pathway), decreased transport of leptin *via* saturable transport pathways across the blood brain barrier to the arcuate nucleus, and possibly protein tyrosine phosphatase 1B inhibition of JAK phosphorylation [51-53]. Whether leptin resistance exists in CKD remains unknown. 

### Evidence for Leptin as a Cardiovascular Risk Factor

Experimental studies suggest a role for leptin as a causative or contributing factor in the pathogenesis of atherosclerosis. Leptin’s proatherogenic effects include the development of hypertension, oxidative stress, endothelial dysfunction, inflammation, platelet aggregation, migration, proliferation and hypertrophy of VSMC. There has been particular interest in the role of leptin in obesity related hypertension. While obesity is associated with central hypothalamic resistance, this phenomenon appears to be selective primarily to its anorectic effect. Chronic hyperleptinemia has been shown to be associated with activation of the sympathetic nervous system (SNS) *via* both central (despite central hypothalamic resistance) and possibly peripheral mechanisms. Elevated leptin levels, either in transgenic mice with hyperleptinemia or by prolonged exogenous administration of leptin, resulted in elevated mean arterial pressure by 10-20mmHg. Administration of α-blockers abolished leptin-induced hypertension, supporting a role for the SNS in the development of hypertension [54, 55]. The stimulatory effect of leptin on the SNS is likely a predominant central effect as transection of distal sympathetic fibers failed to abrogate the hypertensive response [56]. Furthermore, the hypertensive effect of leptin was abolished completely after selective excision of the hypothalamic arcuate nucleus [50]. Leptin has also been shown to directly increase catecholamine production in the adrenal medulla [57]. Additionally, leptin may have direct functional and structural renal consequences in obesity. Acute administration of leptin in rats results in natriuresis and saliuresis independent of GFR, renal blood flow and potassium excretion, an effect that was blunted by blockade of the renal SNS [58, 59]. Of interest, obese rats experienced a diminished diuretic effect in response to leptin [58]. Clinical studies suggest an association between leptin and hypertension. Leptin has been shown to be linked to mean blood pressure in lean subjects with essential hypertension [60]. In addition, there was a strong positive correlation between serum leptin and renal sympathetic activation in men with varying levels of adiposity [61]. However, to date there have been no similar studies in the CKD population.

Observational data support an association between leptin and CVD. Elevated leptin levels have been shown to be associated with early markers of CVD, including increased carotid intima media thickness (IMT) and decreased arterial distensibility [62, 63]. However, other studies have failed to discover a similar association between leptin and carotid IMT [64, 65]. Several studies have highlighted an association between hyperleptinemia and coronary artery disease in various patient populations, including patients with type 2 diabetes and those with hypertension [66-68]. The best evidence to date for leptin’s proatherogenic role comes from the West of Scotland Coronary Prevention Trial (WOSCOPS) [69]. The WOSCOPS study was designed to determine the efficacy of pravastatin in the prevention of ischemic heart disease in men with moderate hypercholesterolemia. In this large prospective study with over 1000 patients and 5 year follow-up, elevated leptin levels predicted the development of new coronary events independent of body mass index and inflammation. Each 1 standard deviation increase in leptin concentrations was associated with a 25% increased relative risk of an event (odds ratio 1.25, 95% confidence interval [CI], 1.10 to 1.43; P<0.001). Increased plasma leptin has also been shown to predict the development of cardiac failure and is positively associated with the severity of left ventricular hypertrophy [70, 71]. Hyperleptinemia is also an independent predictor of acute ischemic and hemorrhagic strokes [72, 73]. 

### Putative Mechanisms for Leptin-Induced CVD 

Experimental studies have provided an insight into the potential mechanistic basis for leptin’s proatherogenic effects. Hyperleptinemia, *via* up regulation of nitric oxide production, resulted in the generation of reactive oxygenation species (ROS). Various studies have shown that leptin-treated mice have elevated levels of various markers of oxidative stress, including malonyldialdehdye, peroxides, isoprostanes and oxidized lipoproteins, a hallmark feature of atherosclerosis, as well as reduction in anti-oxidant molecules, such as glutathione [74-76]. Leptin may also promote endothelial dysfunction, though this remains controversial. The data are conflicting on the potential benefits of leptin-induced nitric oxide (NO) generation and subsequent vasodilatation versus the possible generation of ROS from elevated NO levels [77, 78]. Similar contradictory results have been surmised from clinical studies on the association between leptin, NO and endothelial function, and thus at present the role of leptin remains unclear [65, 79]. Leptin is also associated with increased platelet reactivity and aggregation, enhanced immune function, cardiac hypertrophy, and impaired cardiac contractility [80-83].

### The Role of Leptin in Chronic Kidney Disease

There are sparse and conflicting data on leptin in CKD, particularly its association with CVD and mortality. In general, leptin levels are significantly higher in patients on dialysis, particularly those on peritoneal dialysis, compared to the non-dialysis population [84-86]. This may simply reflect the role of the kidney in the filtration and subsequent tubular degradation of leptin [87]. However, other variables have been shown to affect leptin levels in CKD patients. Teta *et al.* showed that metabolic acidosis reduced the release of leptin from adipose tissue [88]. In addition, uremia reduces leptin gene expression in adipocytes, which may reflect a compensatory mechanism from decreased elimination [89]. In contrast, endotoxins and inflammation in kidney failure have been shown to up regulate leptin production [90]. While some of the previous studies have not reported a correlation between leptin and body mass index, leptin has been shown to be correlated with body fat mass using dual energy x-ray absorptiometry (DEXA) in stage 5 CKD patients [91]. Higher leptin levels correlated with elevated lipid concentration (cholesterol and triglycerides), left ventricular hypertrophy and lower clinical atherosclerosis score in a study involving peritoneal dialysis patients [92]. Leptin has also been shown to be significantly correlated with higher ejection fraction in a group of hemodialysis patients [93]. To date, the largest prospective study of leptin in CKD subjects involved 71 hemodialysis patients with a mean follow up period of 83 months [94]. During the study, 48 (63%) patients died, predominantly due to CVD (67%) and infection (21%). While lower leptin levels were significantly associated with all cause mortality, baseline serum leptin concentrations were also significantly lower in patients who died from CVD (4.7 ± 9.4 µg/L, P<0.05) or infections (4.0 ± 2.7 µg/L, P<0.05). These studies support a role for leptin in the increased cardiovascular mortality of patients with severe kidney failure. The majority of experimental work has shown a positive correlation between leptin and pro-atherogenic effects, whereas an adverse cardiovascular profile in CKD patients in the clinical setting has been attributed to lower levels of leptin. The exact cause for this discrepancy is unknown but low levels of leptin may reflect a state of malnutrition, and hence contribute towards uremic cachexia. Leptin levels are higher in healthier dialysis subjects, and are directly correlated with improved markers of nutrition [92, 95, 96]. Conversely, other studies have reported an inverse relationship between higher levels of leptin and nutritional markers in CKD [91]. In addition, leptin may function as a negative acute response phase protein. In stage 5 CKD patients, CRP was inversely related to negative acute phase proteins, including albumin, transferrin and leptin [95]. However, CRP has also been shown to be positively correlated with leptin in CKD [96]. These clinical studies underscore the difficulty in elucidating the role of leptin in the pathogenesis of CVD in CKD amongst the meshwork of other contributing factors to the CVD burden in this patient population, including nutrition, inflammation and conventional cardiovascular risk factors.

## CONCLUSION

Patients with CKD remain at high risk of premature cardiovascular death. Interventional studies addressing traditional cardiovascular risk factors in this group of patients have failed to yield significant benefits. As such, there has been great interest in alternative cardiovascular risk factors including adipokines. While there has been a preponderance of observational and experimental data linking adipokines to CVD in the CKD and non-CKD population, their exact role remains to be verified. The interplay of other variables associated with uremia further adds to the complexity in determining whether adipokines have significant pro atherogenic properties in CKD. Adipokines like leptin and adiponectin may serve as biomarkers of CVD in CKD. To date, there have been no interventional studies with either adiponectin or leptin on cardiovascular outcomes in the humans, including those with CKD. In conclusion, further studies are necessary to clarify and determine the role of adipokines in CVD in CKD, either as a biomarker or potential therapeutic tool in addressing the cardiovascular burden in CKD. 
